# Gene set based association analyses for the WSSV resistance of Pacific white shrimp *Litopenaeus vannamei*

**DOI:** 10.1038/srep40549

**Published:** 2017-01-17

**Authors:** Yang Yu, Jingwen Liu, Fuhua Li, Xiaojun Zhang, Chengsong Zhang, Jianhai Xiang

**Affiliations:** 1 Key Laboratory of Experimental Marine Biology, Institute of Oceanology, Chinese Academy of Sciences, Qingdao 266071, China; 2 University of Chinese Academy of Sciences, Beijing 100049, China; 3 Laboratory for Marine Biology and Biotechnology, Qingdao National Laboratory for Marine Science and Technology, Qingdao 266237, China.

## Abstract

White Spot Syndrome Virus (WSSV) is regarded as a virus with the strongest pathogenicity to shrimp. For the threshold trait such as disease resistance, marker assisted selection (MAS) was considered to be a more effective approach. In the present study, association analyses of single nucleotide polymorphisms (SNPs) located in a set of immune related genes were conducted to identify markers associated with WSSV resistance. SNPs were detected by bioinformatics analysis on RNA sequencing data generated by Illimina sequencing platform and Roche 454 sequencing technology. A total of 681 SNPs located in the exons of immune related genes were selected as candidate SNPs. Among these SNPs, 77 loci were genotyped in WSSV susceptible group and resistant group. Association analysis was performed based on logistic regression method under an additive and dominance model in GenABEL package. As a result, five SNPs showed associations with WSSV resistance at a significant level of 0.05. Besides, SNP-SNP interaction analysis was conducted. The combination of SNP loci in *TRAF6, Cu/Zn SOD* and *nLvALF2* exhibited a significant effect on the WSSV resistance of shrimp. Gene expression analysis revealed that these SNPs might influence the expression of these immune-related genes. This study provides a useful method for performing MAS in shrimp.

The Pacific white shrimp *Litopenaeus vannamei (L. vannamei*) is a world-wide aquaculture species. After it was firstly introduced to China in 1988, its production has increased rapidly, and it has become the major cultured shrimp species with an annual output value of 7.4 billion US dollars. However, the huge profit is accompanied by multiple challenges like environmental degradation and various shrimp diseases. White spot syndrome virus (WSSV) is regarded as the most severe virus for its wide spread ability and high lethality^1^. Up to now, there is no effective method to prevent the shrimp from WSSV infection. Breeding of WSSV-resistant varieties should be the most effective method to solve the virus disease problem.

As a threshold trait, disease-resistance appears to have low heritability, which is easily influenced by external environments. Marker assisted selection (MAS) is considered to be an effective method in breeding for a complex trait, especially for disease resistance. In order to conduct MAS, markers related to disease-resistance is needed. Association analysis is regarded as a powerful tool for trait-related marker screening^2^,^3^. Through this approach, multiple quantitative trait loci (QTLs) have been successfully identified in some species^4^,^5^,^6^. Genome-wide association study (GWAS) and candidate gene association study are two powerful strategies for association analyses. GWAS has been applied for mapping complex diseases traits in human beings, economic crops and animals^7^,^8^. However, the genome reference sequence is necessary for GWAS. For species without whole genome sequences, the candidate gene strategy was more widely used due to its advantages in specificity and accuracy for association analysis^9^.

With the development of Single Nucleotide polymorphisms (SNPs) genotyping method, a large number of SNPs can be genotyped simultaneously at a lower cost. Recently, SNPs in gene sets based on regulatory networks and gene pathways began to be used for association analyses^10^,^11^,^12^. Gene set based association analyses can eliminate redundant information, improve the efficiency at a lower cost and detect associations with weak effects^13^,^14^. This method has been widely used in human diseases analysis and economic traits studies of animals^11^,^15^,^16^.

In shrimp, innate immune signaling pathways, including Toll pathway, IMD pathway and JAK/STAT pathway were involved in the immune response of shrimp to bacterial or WSSV infection. The immune response of innate immunity includes pathogen recognition, signal transduction and production of effectors. A large number of effectors including antimicrobial peptides (AMPs), antioxidant enzymes, hemocyanin *etc.* are produced to kill the pathogens directly. Both Toll pathway and IMD pathway were responsive to bacteria and virus, and JAK/STAT pathway was involved in WSSV infection^17^. Therefore the key genes involved in the innate immunity were regarded as the basis for disease resistance of shrimp.

The generalized multifactor dimensionality reduction (GMDR) method was developed based on the multifactor dimensionality reduction (MDR) method to be applied in detecting the interactions for gene-by-gene or gene-by-environment^18^,^19^,^20^,^21^. The genes used for GMDR analysis were usually from one pathway which showed similar functions^11^,^15^. It has been used for risk assessment on cancer and other diseases in humans^14^,^22^,^23^. This method was recently introduced for genetic breeding of livestock and poultry^11^.

In the present study, the SNPs in the gene set of the signaling pathways regulating the innate immune response to WSSV infection were selected for association analysis. The GMDR method was used to further explore the influence of gene-by-gene interactions on the WSSV resistance/susceptibility of shrimp.

## Results

### WSSV copy numbers in WSSV-susceptible and WSSV-resistant groups

Before WSSV infection, the average viral load of WSSV per ng DNA of shrimp from the experimental population was 7.46 copies/ng, so the experimental shrimp could be regarded as WSSV free. At 3 days after WSSV infection, the average viral load in dead shrimp in shrimp reached 1.05 × 10^5^ copies/ng DNA. After checking the virus load in shrimp from WSSV-susceptible (sus.) group or WSSV-resistant (res.) group, we found that the viral load in shrimp from sus group was (1.286 ± 0.355) × 10^5^ copies/ng DNA, while that in res. group was (2.793 ± 0.982) × 10^3^ copies/ng DNA. The *P* value from the independent t-test between two groups was 0.001, which means that a very significant difference existed for the viral load between sus. and res. group.

### SNPs validation, selection and genotyping

Validation on the SNPs predicted by bioinformatics analysis was shown in Table S1. The prediction accuracy on the SNPs from Illumina data was 86.5% when the quality score was set at more than 20. The prediction accuracy for SNPs with high quality score (Q > 100) and relative low score (20 ≤ Q ≤ 100) showed no obvious difference. Meanwhile, the parameter of *duel min* was a determinant factor for SNPs prediction with 454 data. The prediction accuracy of SNP loci with parameter *duel min* ≥9 reached 88.2%, while it was not accurate for loci with duel min <9. Thus SNPs with quality score higher than 20 and duel min more than 8 were used as basic data for later SNP screening.

Through bioinformatics analysis, a total of 868 loci from Illumina sequencing data and 776 loci from 454 sequencing data, which were located in the immune related genes, were screened with the Q value higher than 20 for Illumina data and *duel min* higher than 9 for 454 sequencing data. After blasting the related cDNA sequence of these unigenes to the assembled genome sequences established in our lab, 681 loci (593 from Illumina data, 88 from 454 data) with high quality were obtained for further SNPs genotyping analyses in sus. and res. group. Due to the limitations of primer design for the genotyping analysis, a total of 77 SNPs from the 681 loci were chosen for the genotyping based on the SNP annotation and function.

### Population stratification and associations analysis

**Figure 1:**
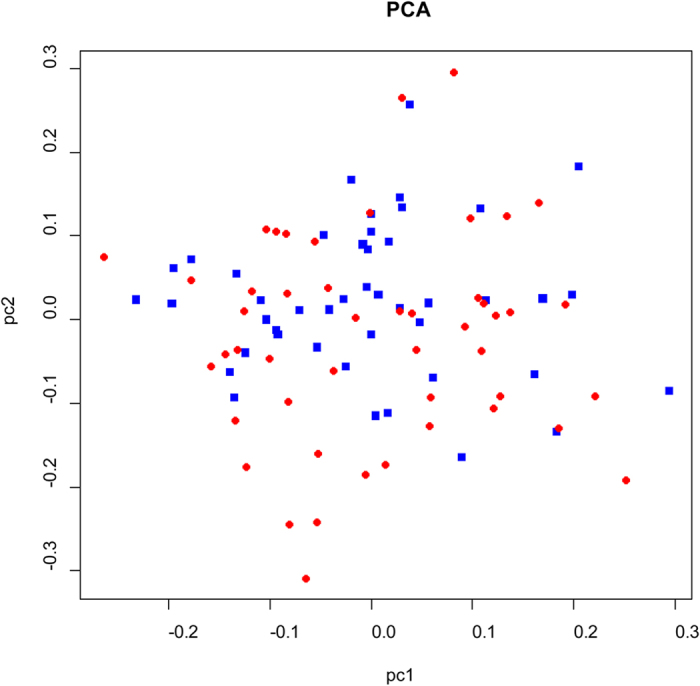
Multidimensional scaling plot for sus and res group. The red dots represented sus group and the blue dots represented the res group.

After quality control, a total of 51 SNPs were qualified for genotyping in 93 individuals of shrimp. The multidimensional scaling analysis (MDS) on these shrimp based on 51 SNPs showed that they were evenly distributed, which indicated that no genetic stratification existed in the studied population (Fig. 1). The genome-wide degree of inflation (λ) was 1.11 and 1.18 for additive and dominance model respectively, which showed that the structure of the studied population had a minor impact on the association analyses.

Under the additive model, three SNPs located in unigene 15411 (*TLR*, sSNP), unigene 16729 (*TRAF6*, nsSNP) and unigene 34129 (*BRAFLDRAFT_123571,* sSNP) were associated with WSSV resistance at a significant level (P < 0.05). Under the dominance model, five SNPs located in unigene 15411 (*TLR*, sSNP), unigene 34129 (*BRAFLDRAFT_123571,* sSNP), unigene 16729 (*TRAF6*, nsSNP), unigene 34569 (*Cu/Zn SOD*, sSNP) and unigene 30237 (*STAM*, 3′ UTR) were significantly associated with the WSSV resistance of shrimp (P < 0.05) (Table 1, Table S3).

### GMDR analysis for SNP-SNP interaction associated with WSSV resistance

**Figure 2:**
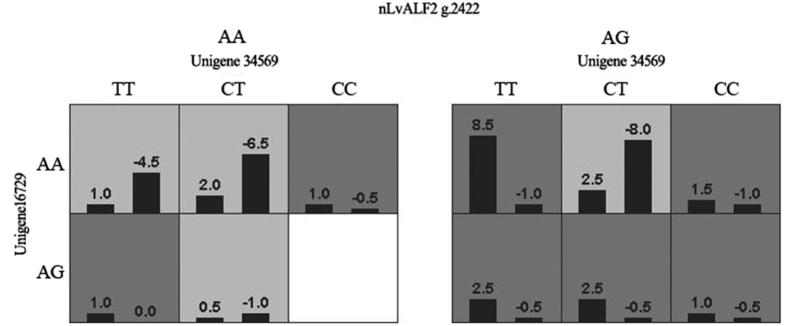
The analysis results of best three-factor model. When the sum of scores (numbers above bars) in one cell is above or below zero, the cell will be categorized as ‘high risk’ or ‘low risk’. High-risk groups are marked with dark grey, and low-risk groups are with light grey. The white cell is empty. The left bar and right bar represent susceptible individuals and resistant individuals, respectively. Genotypes of nLvALF2 g.2422 were AA and AG, and genotypes of Unigene 34569 were TT, CT and CC. The locus in Unigene16729 contained two genotypes AG and AA.

All loci showing associations with WSSV resistance identified in this study and those in our previous study^24^,^25^ were used for GMDR analyses to detect the combined effects. The best interaction models from two-loci to four-loci were shown in Table 2. Combination of three SNPs (Unigene34569, Unigene16729 and *nLvALF2* g.2422) exhibited significant association with resistance/susceptibility of shrimp to WSSV. The interaction among above three SNPs were shown in Fig. 2, in which the risks of shrimp to WSSV infection caused by different combinations of SNPs were displayed. Different combinations of genotypes were classified as ‘high risk’ (hr) group and ‘low risk’ (lr) group. Individuals with low risk combinations are supposed to be more difficult to be infected than those with high risk combinations. Therefore, shrimp with the combination of AA-TT-AG, AA-CC-AA, AG-TT-AA, AG-TT-AG, AG-CT-AG, AG-CC-AA, AG-CC-AG in *nLvALF2* g.2422, Unigene34569, Unigene16729 were supposed to be susceptible to WSSV, while shrimp with the combination of AA-TT-AA, AA-CT-AA, AA-CT-AG, AG-CT-AA were resistance to WSSV.

### Verification of GMDR analysis

**Figure 3:**
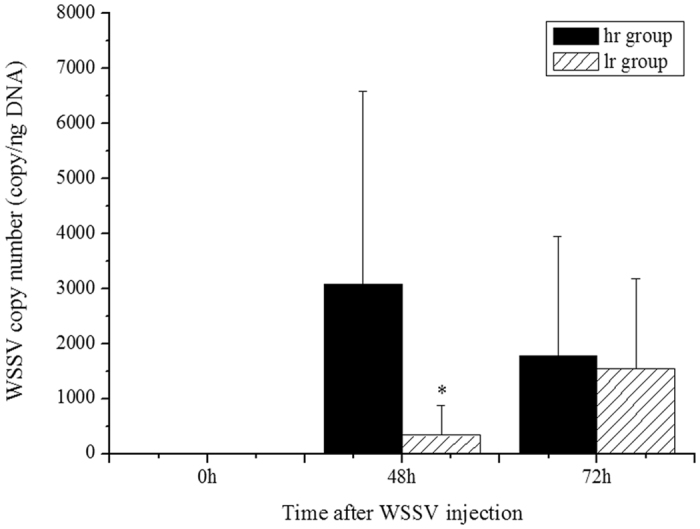
The comparison of the viral copy number between hr group and lr group at different time after WSSV injection.

Before WSSV injection, the viral load of WSSV in shrimp were around zero, which indicated that the shrimp was WSSV free. The viral load in shrimp from the ‘high risk’ (hr) group was much higher than that in shrimp from the ‘low risk’ (lr) group at 48 hpi, However, no obvious difference was observed between these two groups at 72 hpi (Fig. 3).

**Figure 4:**
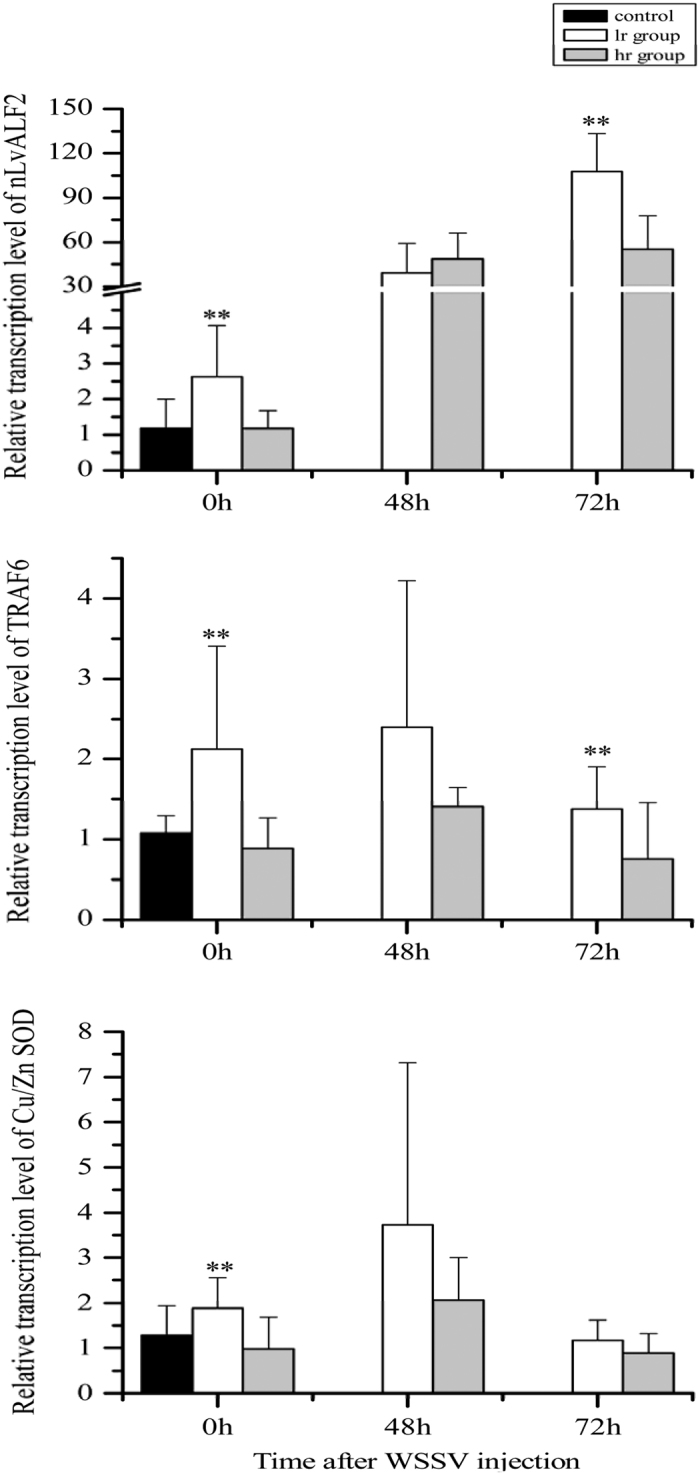
The expression profiles of *nLvALF2, TRAF6* and *Cu/Zn SOD* in hr group and lr group after WSSV infection. The mean of hr and lr group at 0 h was set as a control group. Asterisks indicate significant differences (*P < 0.05; **P < 0.01) between hr and lr group at the same time.

The expression levels of Unigene 34569 (*Cu/Zn SOD*), Unigene 16729 (*TRAF6*) and *nLvALF2* in hr group and lr group showed significant differences (*P* < 0.01) (Fig. 4) in shrimp without WSSV infection. During WSSV challenge, the gene expression levels of Unigene34569 (*Cu/Zn SOD*), Unigene16729 (*TRAF6*) in lr group were higher than those in hr group at 48 h after infection, while no difference was detected for *nLvALF2* at this time. At 72 h after WSSV infection, the expression levels of *TRAF6* and *nLvALF2* in lr group were higher than those in hr group, while no difference was detected for the expression level of *Cu/Zn SOD* between these two groups.

## Discussion

SNPs are the most abundant and widely-distributed mutations in genomes. They can be divided into coding-region SNPs (cSNPs), perigenic SNPs (pSNPs) and intergenic SNPs (iSNPs) based on their positions in the genomes. As the SNPs may directly influence the gene translation or gene transcription, they were widely used as molecular markers for disease genetics and pharmacogenomics studies^26^. The application of SNPs in association study for quantitative trait locus (QTL) mapping also have been attracting more interests^27^,^28^,^29^. As enormous transcriptome data were produced by next generation sequencing, high throughput SNPs can be easily obtained by bioinformatics prediction. In our previous study, a total of 96,040 SNPs were predicted from two transcriptomes of *L. vannamei*. In order to reduce the false-positive rate of SNPs prediction and obtain the most accurate SNPs, we firstly analyzed the filtering parameters for different sequencing methods in the present study. The SNPs prediction accuracy was up to more than 86% when a high Q score (for Illumina sequencing data) and *duel min* score (for 454 sequencing data) were set^30^.

Shrimp immune related genes were involved in the defense of shrimp against WSSV infection^31^. In the present study, 77 loci located in the immune related genes were chosen as candidate SNPs for WSSV resistance analyses. The JAK/STAT signaling pathway plays key roles in the antiviral immunity^17^. In the present study, one locus on unigene 30237 (*STAM*, 3′ UTR SNP) was associated with the WSSV resistance of shrimp both in additive and dominance model (P < 0.05). STAM (Signal transduction adaptor molecule), as a key gene in the JAK/STAT signaling pathway, was directly interacted with JAK to mediate the signal transduction and promote the endocytosis of WSSV^32^. It was reported that SNPs located at the 3′ UTR of the gene could alter the gene transcriptional efficiency through affecting the combination between mRNA and the regulatory elements^33^,^34^. As the identified locus located at 3′ UTR of *STAM*, the SNP in unigene 30237 (*STAM*) which showed association with WSSV resistance might affect the transcriptional efficiency of *STAM*.

The SNPs located in unigene 16729 (*TRAF6*, nsSNP) and unigene 15411 (*TLR*, sSNP) showed significant association with WSSV-resistance (P < 0.05). TLR are the upstream factor in Toll pathway, and *TRAF6* can regulate the activation of NF-kB and various stress kinases^35^. *TRAF6* showed up-regulation at the transcriptional level after WSSV infection, and could activate the expressions of AMPs in *L. vannamei*^36^. The detected SNP in *TRAF6* was a nonsynonymous mutation which changed the amino acid sequence. Considering the crucial function of *TRAF6* in Toll pathway, this nonsynonymous SNP may play important roles in shrimp to defend WSSV infection.

One SNPs located in genes encoding immune effectors also showed association with WSSV-resistance. Shrimp with allele C at locus 389 of Unigene34569 (*Cu/Zn SOD*, sSNP) showed resistance to WSSV. This SNP was a synonymous SNPs. Although the synonymous SNPs can not change the protein structure directly, it has been proved that sSNP can affect the biological process by the following three mechanisms. One was that sSNPs and some functional nsSNPs might be in strong linkage disequilibrium, and the second was that allele-specific differences in mRNA folding might influence the splicing, transcription and regulation. The third explanation was that the codon usage bias can affect the protein translation and folding.

As mentioned previously, multiple molecules in the humoral and cellular immune system take part in defending against WSSV. Compared to one gene, mutations in gene pathways could explain most phenotypic variance for certain traits^16^,^37^. That’s why we chose lots of SNPs from immune gene pathways in this research. Through this strategy, more potential genes or SNPs which might affect the WSSV-resistance could be found.

In some ways, development in human researches can greatly facilitate the study in animals or non-model species. Although thousands of SNPs were detected to be associated with various human diseases, most of them can only explain part epigenetic changes of complex disease^38^. As we know, the biological processes are usually accomplished by the interactions among lots of molecules or pathways. Investigation on the contribution of functionally relevant gene-gene (SNP-SNP) interaction and gene-environment interaction to complex diseases has been proved to be useful in many cases^11^,^13^,^21^,^22^. Statistical method including logistic regression models, multi-loci linkage disequilibrium (LD) tests were developed to analyze a large sample size data^39^. Using unconditional multiplicative logistic regression method, researchers have found the interaction of SNP genotypes in several mismatch repair genes was associated with risk for breast cancer^15^.

MDR method was the primary tool for studying genes or SNPs interaction network for moderate sample size data^18^. Different versions like OR-MDR, GMDR, MB-MDR were created for different purposes^19^,^40^,^41^. Based on the GMDR analysis, several SNPs as well as SNP-SNP combination from the IGF1/FoxO signal transduction pathway were reported to be associated with economical traits in pigs^12^. The present study applied the GMDR method in shrimp WSSV resistance association analysis for the first time. The loci in *LvALFs* related to WSSV resistance of shrimp found in our previous studies were also involved^24^,^25^. In the present study, a three-factor model consisted of loci from unigene34569, unigene16729 and *nLvALF2* showed significant association with viral resistance. According to GMDR analysis results, lr group and hr group were divided based on the genotyping result of above three loci. The average WSSV copy numbers in hr group was apparently higher than that in lr group at 48 h after WSSV infection, which suggested that lr group might have higher WSSV resistance than hr group. When we compare the expression level of unigene34569, unigene16729 and *nLvALF2* in lr group and hr group, we found that the three genes in lr group showed higher expression levels than those in the hr group. These data indicated that the high expression of these genes might affect the replication of WSSV. Further work needs to be done to validate the combination of SNPs related to WSSV resistance of shrimp in a larger population and investigate the feasibility of these markers in shrimp breeding.

## Conclusion

This study presents the first gene set based association analyses for WSSV resistance in *L. vannamei.* A total of 5 SNPs in these immune related genes were detected to be associated with WSSV resistance. The marker combinations associated with WSSV resistance were also identified through SNP-SNP interaction analysis. These results were useful for understanding the genetic basis for disease resistance in shrimp and will assist the selective breeding of shrimp with WSSV-resistance.

## Materials and Methods

### Experimental animals for association study

The shrimp individuals used for association analysis were generated from multiple families of Kehai No. 1 variety, which was a new selective breeding variety in China^42^. Five hundred healthy shrimp, with an average body length of 6.18 ± 1.11 cm and body weight of 3.59 ± 1.57 g, were cultured in filtered sea water at 24 °C with continuous aeration in plastic tanks. The shrimp were reared without feeding for 2 days. Then the shrimp were fed with WSSV infected shrimp tissue (average 10^5^–10^6 ^copies/ng DNA) four times (2 times per day) to simulate the normal infection process during shrimp culture. Every shrimp was checked to ensure that they ate shrimp tissues infected with WSSV. The shrimp which did not eat would be fed with shrimp tissues in the second day. After the infection, the shrimp were fed with artificial food pellets twice a day. All tanks were checked daily and dead shrimps were collected and marked. On the 16^th^ day after artificial infection, the number of live shrimp was stable. Forty eight survived individuals on 19^th^ day were regarded as WSSV-resistant samples (res group), and forty eight individuals died at the beginning after inoculation were regarded as WSSV-susceptible samples (sus group). All experimental materials were stored at −20 °C for DNA extraction and virus detection.

### SNP validation and selection

SNPs were predicted based on several transcriptomic data sets through Illumina Hiseq 2500 and Roche 454 sequencing^30^. According to annotations, all SNPs in 49 unigenes predicted from Illumina data and in 7 unigenes predicted from 454 data related with JAK/STAT, TOLL and IMD pathways were picked out. SNPs with quality score higher than 20 were chosen for further analysis. In order to analyze the accuracy of predicted SNPs, we randomly selected 43 loci from Illumina data and 32 loci from 454 data for validation by PCR method (Table S2).

After validation, SNPs with high quality were selected and the genomic sequence of selected SNPs were obtained by blasting the unigenes’ cDNA sequences to the genomic database of our lab.

### SNP genotyping

Selected SNPs located in the immune related pathways were genotyped in WSSV susceptible/resistant groups using ABI Prism SNaPshot™ Multiplex System. For SNaPshot genotyping, the SNP must satisfy the condition that no other SNPs existed in the upstream and downstream sequence of the SNP within 150 bp. Therefore, 12 SNPs located in the genes involved in JAK/STAT pathway, 19 SNPs in TOLL pathway related genes, 12 SNPs in IMD pathway related genes, and 34 SNPs located in other immune genes were genotyped (Table 3). The SNP information were submitted to NCBI dbSNP with submitted SNP (ss) number from 2137123241 to 2137123317. The SNP genotyping was conducted by Shanghai MapBiotech Company Limited. For easy to describe, the name of the unigenes used in later part represented the testing SNP locus in it.

### Data analysis

Quality control of genotyped SNPs was performed using “*check.marker*” function in GenABEL package^43^. Those SNPs with low Minor Allele Frequency (MAF), low call rate and deviation to Hardy-Weinberg Equilibrium were dropped prior to main analysis. Multidimensional scaling were implemented on filtered SNPs to assess the population stratification in GenABEL package. As the phenotype is binary, we performed association analysis using logistic regression method under an additive and dominance model, the body weight of each shrimp was used as covariate. This analysis was accomplished by “*mlreg*” function in GenABEL package. The genome-wide degree of inflation (λ) was calculated to test for any hidden substructure, and the final P-value was corrected by inflation factor. Associations were deemed statistically significant at the 5% empirical significance cutoff.

In order to know the potential interactions among SNPs located in different genes, SNPs associated with WSSV resistance with statistical significance (P ≤ 0.05) were chosen for further gene-gene interaction analysis. Meanwhile, SNPs located in *nLvALF1* and *nLvALF2* (loci *nLvALF1* g.1370, g.1419 and *nLvALF2* g.2422, g.2466, g.2529) which was proved to be potentially associated with WSSV resistance in previous reports^24^,^25^ were also chosen for the interaction analysis. Through the cross-validation strategy, GMDR divided various combinations of genotypes into ‘high’ or ‘low’ risk genotypes^19^. In the present case, the high risk means more susceptible to WSSV, and the best two-, three-, and four-factor (SNP) models were given. Shrimps with ‘low’ risk genotypes were considered to be high resistant to WSSV than those with ‘high’ risk genotypes. Models were considered to be significant at *P* < 0.05.

### Validation of SNPs interaction model

**Figure 5:**
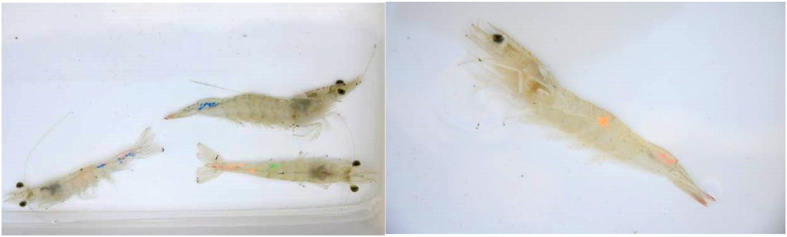
Shrimps labeled with different fluorescent dyes.

Two hundred health shrimp carrying no specific pathogens were chosen to verify the effect of gene-gene interaction based on the best three-factor model given by GMDR analysis. Firstly, we separated shrimp into two groups (100 per group), then each shrimp was labeled with different combinations of four fluorescent dyes (green, red, blue, orange) (Fig. 5) in different part of the body. After marking, two pleopods from each shrimp were taken to extract DNA for SNP examinations. Shrimp were then reared in seawater with continuous aeration.

Genomic DNA extracted from the pleopods of each shrimp were used for SNPs genotyping by Sanger sequencing. The primers used for genotyping were shown in Table 4. All PCR products were sent to Beijing Genomics Institute for sequencing. Shrimps with predicted best combinations were grouped as ‘low’ risk (lr) group and vice versa. There were forty individuals in lr group and hr group, respectively. Each shrimp was injected with 10^4^ copies of WSSV (dissolved in PBS) at the last abdominal segment (set as 0 h) following the method described previously^24^. Five or six shrimp were collected at 0, 48 and 72 h after WSSV injection (hpi) from lr group and hr group, separately, and these shrimps were preserved in liquid nitrogen for further gene expression analysis. Shrimp died during the experiment were also collected for virus check.

The swimming legs were picked to extract DNA for quantification of viral load. Total RNA was extracted from cephalothoraxes. The procedures for RNA and genomic DNA extraction were the same as those described previously^24^. The cDNA synthesis was performed using PrimeScript RT Reagent kit with gDNA Eraser (TaKaRa, Japan). The virus load in each shrimp was checked according to the method described by Sun *et al*.^44^. Transcriptional levels of the three genes referred in the best three-factor models, including unigene34569, Unigene16729 and *nLvALF2* were analyzed by real-time qPCR (RT-qPCR). 18 S rRNA was used as a reference gene. Relative expression levels were calculated by 2^−ΔΔCt^ method. The average transcriptional level of the genes in hr group and lr group at 0 h were used as controls. All these data were analyzed by independent samples *t-test* with a significance level of *P* = 0.05 using SPSS 16.0.

## Additional Information

**How to cite this article**: Yu, Y. *et al*. Gene set based association analyses for the WSSV resistance of Pacific white shrimp *Litopenaeus vannamei. Sci. Rep.* **7**, 40549; doi: 10.1038/srep40549 (2017).

**Publisher's note:** Springer Nature remains neutral with regard to jurisdictional claims in published maps and institutional affiliations.

## Supplementary Material

Supplementary Information

## Figures and Tables

**Table 1:** Results of significantly associated SNPs with WSSV resistance from the additive and dominance models.

SNPs	Additive model			Dominance model		
	*chi*^*2*^	*P-value*	*Pc-value*	*chi*^*2*^	*P-value*	*Pc-value*
Unigene15411	6.124	0.013	0.019*	6.173	0.013	0.022*
Unigene16729	5.007	0.025	0.033*	5.007	0.025	0.039*
Unigene34129-1	4.834	0.028	0.037*	5.789	0.016	0.027*
Unigene34569	1.397	0.237	0.261	4.911	0.027	0.041*
Unigene30237	3.961	0.047	0.058	4.808	0.028	0.043*

chi^2^: Chi-squared test value of each SNPs under different models.

*Pc-value:* P-value corrected for inflation factor.

**Table 2:** Best gene-gene interaction models of immune genes by GMDR.

The best model	Test accuracy	*P* value	CVC
Unigene34569 & nLvALF2 g.2422	0.664	0.055	7
Unigene34569 & Unigene16729 & nLvALF2 g.2422	0.693	0.011*	6
Unigene34129-1 & Unigene34569 & nLvALF1 g.1419 & nLvALF2 g.2422	0.685	0.055	7

**Table 3:** SNPs in the immune related genes for genotyping.

Annotation	Unigene	Locus	Allele	
PI3K1	Cl1819_Contig1	3941	G/A	
PI3K6	Unigene19157	654	A/G	
SOS1	Unigene11468	1316	A/G	
SOCS2	Unigene15654-1	542	A/G	
	Unigene15654-2	1004	C/T	
	Unigene15654-3	1445	A/G	
SOCS7	Unigene18924-1	1578	T/A	
	Unigene18924-2	2148	C/T	
	Unigene18924-3	2802	G/C	
CBL	Unigene30068	539	T/C	
STAM	Unigene30237	792	A/G	
PIAS	Unigene30238-2	1574	G/A	
Spatzle	Unigene4117	486	G/A	
Pelle	CL3068.Contig1-1	182	T/C	
	CL3068.Contig1-2	257	A/G	
Pelle	Unigene4617	1592	C/T	
Toll	Unigene23477-1	878	A/C	
	Unigene23477-2	2251	T/C	
Toll	Unigene22832	378	G/C	
Toll2	Unigene350-1	2016	T/C	
	Unigene350-2	2530	G/A	
Toll3	Unigene4407	365	T/C	
TLR	Unigene11581-1	176	T/C	
	Unigene11581-2	2418	A/G	
TLR	Unigene15411	2742	G/A	
TRAF6	Unigene16729	1779	A/G	
TLR	Unigene15094	1569	A/T	
TLR 3	Unigene20289	1461	T/C	
TLR 3	Unigene3989	509	A/C	
TLR 3	Unigene33797	898	A/G	
Dorsal	Unigene26443	1652	G/A	
Cactus	Cl1355_Contig1	615	T/C	
	Cl1355_Contig2	511	A/T	
Relish	CL2370.Contig1-1	141	G/T	
	CL2370.Contig1-2	371	T/C	
	CL2370.Contig4	235	A/G	
Relish	Unigene26310-1	1862	C/T	
	Unigene26310-2	2309	T/G	
	Unigene26310-3	2541	G/C	
IAP2	Cl3323.Contig3-1	295	G/C	
	Cl3323.Contig3-2	853	T/C	
TAB	Cl498.Contig2 -1	102	A/C	
	Cl498.Contig2-2	161	A/G	
ACP	Unigene2052	829	A/G	
AKP	Unigene4337	393	T/C	
AKP	Unigene16582	734	C/G	
AV	Unigene22907	2170	A/G	
AV	Unigene35531-1	442	T/C	
AV	Unigene35531-2	596	G/T	
AV	Unigene8890	872	T/C	
C Lectin 7	Isotig05963	583	C/G	
Crustin	Cl3250.Contig1	104	C/G	
Crustin	Cl5034.Contig2	126	A/G	
Crustin	Unigene6833	329	A/G	
Crustin2	Unigene18922	362	T/A	
Crustin4	Unigene6804	174	A/G	
Hemocyanin	Cl438.Contig2-1	212	G/T	
Hemocyanin	Cl438.Contig2-2	334	C/A	
Hemocyanin	Cl438.Contig8	433	C/T	
Hemocyanin	Unigene13435	736	T/C	
Hemocyanin	Unigene26970	450	A/C	
Hemocyanin	Cl1313.Contig3	999	T/C	
HSP	Unigene779-1	1190	T/A	
HSP	Unigene779-3	2561	A/G	
HSP2	Cl4154.Contig2	363	T/C	
Peroxidase	Unigene15749	1783	G/C	
Peroxidase	Cl2968.Contig2-1	702	A/C	
Peroxidase	Cl2732.Contig2-1	3234	A/G	
Peroxidase	Cl2732.Contig2-2	1090	C/A	
Peroxidasin	Cl6026.Contig1	1181	T/A	
Peroxiredoxin	Unigene9678-1	439	A/G	
Polyubiquitin	Cl369.Contig1	805	A/G	
Cu/Zn SOD	Unigene34569	389	T/C	
ProPO	Unigene11847	1993	C/T	
ProPO	Unigene27524	5760	C/G	
BRAFLDRAFT	Unigene34129-1	201	T/C	
	Unigene34129-2	899	T/C	

**Table 4:** Primers used for SNPs genotyping (labeled with G) and gene expression detection (labeled with RT).

		Primer sequence (5′–3′)	Tm (°C)
nLvALF2 (G)	Forward	ACTAACCCTTTCGCTCCCACCCAC	56
	Reverse	TATTGGATGAGGTATCAACATTCGC	
nLvALF2 (RT)	Forward	GGCAACTGTATTTCAGGGGTCG	54
	Reverse	CTGCGTGTCGTTCTTCTTCG	
TRAF6 (G & RT)	Forward	CTGACCCTTTAGTGGACGCAT	57
	Reverse	AGGTTCCTGTGCTGGGTTGA	
Cu/Zn SOD (G & RT)	Forward	TTGGACTTCCACCGCCAC	57
	Reverse	TCCAGTCCAGGGAAATGTGC	
18 S rRNA	Forward	TATACGCTAGTGGAGCTGGAA	55
	Reverse	GGGGAGGTAGTGACGAAAAAT	
