# Supplementary material for: Gene set based association analyses for the WSSV resistance of Pacific white shrimp Litopenaeus vannamei
Source: Sci Rep. 2017 Jan 17;7:40549. doi: 10.1038/srep40549 (PMC5240139; doi:10.1038/srep40549)
Supplement: Supplementary Information [file srep40549-s1.pdf]

Gene set based association analyses for the WSSV resistance of Pacific white shrimp *Litopenaeus vannamei*

Yang Yu[1+], Jingwen Liu[1,2+], Fuhua Li[1,3]*, Xiaojun Zhang[1], Chengsong Zhang[1]， Jianhai Xiang[1,3]

[1] Key Laboratory of Experimental Marine Biology, Institute of Oceanology, Chinese Academy of Sciences, Qingdao 266071, China.

[2] University of Chinese Academy of Sciences, Beijing 100049, China

[3] Laboratory for Marine Biology and Biotechnology, Qingdao National Laboratory for Marine Science and Technology

[+.] Contributed equally to this manuscript.

* Corresponding author:

Fuhua Li

Address: Key Laboratory of Experimental Marine Biology, Institute of Oceanology, Chinese Academy of Sciences, Qingdao 266071, China

Tel: 86-532-82898578

Fax: 86-532-82898578

Email: fhli@qdio.ac.cn

Supplementary Table S1. Validation on the predicted SNPs from Illumina and 454 transcriptome data. N represented no polymorphism existed, and Y indicated the polymorphism existed.

	Illumina data				454 sequencing data		
	SNP	Q value	Existence (Yes/No)		SNP	duel min	Existence (Yes/No)
Unigene35531	G/A	999	N	isotig05512	A/G	9	N
	A/C	107	Y		A/G	15	Y
	C/T	29.3	Y		T/C	11	Y
	T/C	999	Y		C/T/A	18	Y
	T/G	44.4	Y		A/G	11	Y
Unigene26443	A/G	120	Y	isotig03572	C/T	2	N
CL2370.Contig1	G/T	68.3	N		G/A	2	N
	T/C	50.9	Y	isotig01785	C/T	4	N
	C/T	999	N		A/G	12	Y
CL2370.Contig4	A/G	36.7	Y		A/G	10	Y
Unigene26310	T/G	155	Y	isotig01666	T/C	18	N
	G/C	999	N		A/G	18	Y
CL1355.Contig1	T/C	153	Y	isotig13143	A/G	2	N
CL1355.Contig2	A/T	38	Y	isotig06076	T/C	8	N
	C/A	999	Y		T/C	8	N
Unigene30068	T/C	999	Y	isotig00366	C/T	7	Y
Unigene30237	T/C	999	N		C/T	7	Y
	T/A	141	Y		G/A	2	N
	A/G	36.3	Y		C/A	10	Y
	A/G	139	Y	isotig10652	G/A	6	N
CL2993.Contig4	A/G	999	Y		T/C	6	N
	A/T	999	Y	isotig00473	A/T	3	N
Unigene37840	C/T	111	Y		C/T	3	N
	A/G	999	Y		A/G	2	N
Unigene11468	A/G	999	Y	isotig00237	T/G	9	Y
CL1819.Contig1	A/G	143	Y		G/A	9	Y
Unigene19157	A/C	999	Y		C/T	9	Y
Unigene15654	A/G	143	Y	isotig01060	C/T	9	Y
	C/T	999	Y		C/A	7	N
	A/G	999	Y		C/A	7	N
Unigene18924	T/C	999	Y	isotig02283	C/A	3	N
	T/C	999	Y		A/G	3	N
	C/G	104	Y	isotig00239	T/G	12	Y
Unigene34129	C/T	999	Y		C/T	12	Y
Unigene9058	C/T	999	Y	isotig05737	T/C	14	Y
CL1819.Contig1	A/G	143	Y				
Unigene19157	A/C	999	Y	**duel min≥9**	**accuracy 0.882**		
**Q value>20**	**accuracy 0.865**			**duel min < 9**	**accuracy 0.111**		

Supplementary Table S2. Primers used for SNPs validation.

**Illumina** Unigene		Primer	Tm	SNP number
Unigene35531	F	ACAGCCGTGGCAACCTTATA	54	5
	R	CCCTCGTGGTTCTCATCACT		
Unigene26443	F	GCAGTAGGGGATGGTTTTGC	51	1
	R	GTTGACTGGAGGGTGTACCA		
CL2370.Contig1	F-1	AGGGCTGGAGAACACAAGTT	52	3
	R-1	TTCACACTCAGAGCGTGCAA		
	F-2	ACACGTATATACTGCTGGTTGC	51	
	R-2	AGTGGTCTGAGTATGGAGAAGC		
CL2370.Contig4	F	TTCGAGGTGGGATGAGTTCC	55	1
	R	AGCTGGTCCGTGATTCCATT		
Unigene26310	F-1	GCTGACCCCAATGCCAAAAA	56	2
	R-1	CCGGGCCATCTGAATACTGT		
	F-2	CCACTACATTCTGCCCTTGACTC	56	
	R-2	ACAGCGACGATAATAATCGGTCT		
CL1355.Contig1	F	GTGAAAACTAACTGTGGTCCGTG	54	1
	R	TTATGATGACGTTGTCTTCCAATT		
CL1355.Contig2	F	TCATACTACGATTCCCACAACGA	56	2
	R	GCTGAGTGTCTCCGTCTTCATCT		
Unigene30068	F	AAGTCATGTTTGTTTGCTAGTTGGC	58	1
	R	AATAGCATGGCATAGCAGTGAATGT		
Unigene30237	F-1	GCGAGTGAGTGAAAGAGTGC	50	4
	R-1	GCATGTGACGTTGAGACACT		
	F-2	TCTTACACGAAGACAGGATGAGATA	54	
	R-2	TTTTGTTCCCAGTAGAGTCAGAGTT		
CL2993.Contig4	F	TACCACACACACCCTGTTGG	53	2
	R	CTGTTCTTCTTTGTGTCCACTTCA		
Unigene37840	F-1	CTCCCAGATACCTGAACATCTTGTG	58	2
	R-1	GTCCCCAAAAGTATGAGGAAAACAG		
	F-2	AGTAGCAGCATCAAAACACTGGAGA	58	
	R-2	TCGGACTTATGAAGAATGCAAAATG		
Unigene11468	F	AGGTGTTTTGTGACCAGCTTC	53	1
	R	GTATACATCAAACTCTGCACCCTCT		
CL1819.Contig1	F	AAACAGAGGATACTCACTGGCACGTT	59	1
	R	CTATAGGGCGGAATACTGGGAAAAG		
Unigene19157	F	AGACAAACTTGGGACTATGGGTATC	56	1

	R	GGGCGATTAAACCACAGTCC		
Unigene15654	F-1	AGTTTGGACGCTGAGAGAATAGAG	56	3
	R-1	TCGCCGGACACGAAGATCAA		
	F-2	CATTGTGTGGCAGGCTCAGA	56	
	R-2	CAAGCAACAACGATGCCTCTG		
	F-3	CACCTTAACAGGAAAAGTAAGATCC	53	
	R-3	CCGACAAACAAAGCTATTAAGATAAG		
Unigene18924	F-1	TTGGAATTATCTAAATCCTGGTGAG	53	3
	R-1	CGACTGAAAGACTATTTGTGCTCCC		
	F-2	TTTTCAATGATGGAGGCAAAGTCG	55	
	R-2	TCCAATGGCATTAACACTGGATTG		
	F-3	AATGAGTCATGGGAGAAGGAGAG	56	
	R-3	GAGGGTTGTGTGTGGATTGATAAG		
Unigene34129	F	CTCTTCTGTGGAGGTTCTTTAGCCC	55	1
	R	GGTATTATGCCCCTTCATAAATACA		
Unigene9058	F	TTTACCTGTGTTTTACCAAGCCAC	55	1
	R	CAATCTGTTTAATGGCCACAGGAA		
CL1819.Contig1	F	AAACAGAGGATACTCACTGGCACGTT	60	1
	R	CTATAGGGCGGAATACTGGGAAAAG		
Unigene19157	F	AGACAAACTTGGGACTATGGGTATC	56	1
	R	GGGCGATTAAACCACAGTCC		

**454** Unigene		Primer	Tm	SNP number
isotig05512	F	GTCGTTCTTCTTCGCCTTAACTATT	57	5
	R	AGAGCTGGAGCTGTTAGGACACTAC		
isotig03572	F	AGGGAGACACCGAACTCTTCACAAC	60	2
	R	ATCGAATACGACTCCCTCAATGTCC		
isotig01785	F	GAGCTTTGGATCATTCCAGTCCTTC	60	3
	R	AGCATTCTCATAACGCTCATTCACG		
isotig01666	F	TCTCTGTTGGAGGCCTAGATGA	55	2
	R	AATATTCCGAGCACACCCTGAC		
isotig13143	F	CACCCCTTCTTGGAAGTCCA	53	1
	R	ACATGGCGAGGGTGTATGTT		
isotig06076	F	GATGAAATAGGAGGGGAACTGTACT	55	2
	R	TTTATTCCCCTTTCTATTCAAGCAC		
isotig00366	F	GTAATGAGGCACTCATGCTTTTCGA	60	4
	R	ATTGGACAGACGTTCAGCATCAAAT		
isotig10652	F	TGCCAGCTGAACAGTGAACT	52	2

	R	GAACCAAGTGCGGGCAATAC		
isotig00473	F	CCAGCTCAGCAGAGATCCTT	52	3
	R	CAAAAATTGGTGGGTTGAGCA		
isotig00237	F-1	TAGCAGCACCATACCGCACAT	57	3
	R-1	GGGCAACAGGATCAAGCAGAC		
	F-2	ACACGGTTCTGGTGTGTCTT	50	
	R-2	GCCAAGGCCTTCGAACTACT		
isotig01060	F	AAACTGAAATGGCAAAATGCG	52	3
	R	GGTGATGGGAATATGGGACTC		
isotig02283	F	CGAGTACATCATCAACTGTGCC	55	2
	R	CCTTTTAAACGGACTTGGCGAA		
isotig00239	F	CGCAGGATTCTTCGAAGCAG	56	2
	R	GAAACGCGACAAGGGTCTTC		
isotig05737	F	GCATGTTCTATTTCAACGATCATAA	55	1
	R	GGATTTCATTTCCTTTCATTTCA		

Supplementary Table S3. SNP information of significant markers identified in association analysis.

Marker ID	SNP position and flanking sequence
Unigene15411	GAAGAGAAGCTCTACGCTGTCTGGCACCGAAGACGCGCTCAGGCTCTCCAGCTGGTTGTGGCTCACGTCGAGGGTCCTCAGCTCCAGCTTGCTCTCCA[A/G]ACTGAAGTAGTTGCCAAGGTCGCGGATTCTGTTGTTGTGGAGGTCAAGGTACTGCAGCTGATCGGGGATGAGCGCGTAGTCGAACCACGAGATCCTGT
Unigene16729	TTTTGACTCTATTTATTCATGGAATGCAAAGTGAGAATGATGACTTTCTTGACTGGCCCTTTAGTGGACGCATAACACTTTCTGTACTAGACTGTGAT[G/A]TATCATTACCTAAAAACCATATCACAGAGACCATGGTGACAAAACCAGGTCTGCAGGCATTCAAGCGTCCTGATGTTGCTCGTAATCCAAAAGGATTT
Unigene34129.201	AAAAAGGTAAACCATCCTGCTGTACAGGCTTTAGCCAAACAGCTAGACCTGCAGCACACATTTGGGTTTATTAAACTTGTGGCTTTTCCTGCTGCGGA[T/C]ACTCCTGGTGTCAATGCGATGACTGCCGCCGAGATCACAACAGGGATGCCCCAAGGGTCAACCAGCACCCCTGGCCGGGCTTTCATTAACAGCAGCTC
Unigene34569	GAAAAACCACGGCAGCCCCTTGGACTTCCACCGCCACGCCGGCGACTTGGGCAACGTCATCGCCGACTACAACGGCGTGGCTCGCATCTCCTTATT[C/T]GACAGGCACATTTCCCTGGACTGGAACTCTCCGGTATACATCGGCGGGCTCGCCTTCGTCATCCACGCCGGCGAGGA
Unigene30237	ATATGAAGTTAATTACTTATGAATTATTTCTCAGTTCAGATAAAAAAGTTTCACAGAAAGTTAGCTGAATTTACACTGGTAATCCTCTGCCTAGTTAT[A/G]ATCATCTGCCATTCTGATCCCAACAAATCAAGGTGCAAAAATTTCTTTGGATACAGAGGCTGCAACACTGGGATTGGCTAGTACAAGGAACATCTACT
